# A Novel Likely Pathogenic Variant in the *BLOC1S5* Gene Associated with Hermansky-Pudlak Syndrome Type 11 and an Overview of Human BLOC-1 Deficiencies

**DOI:** 10.3390/cells10102630

**Published:** 2021-10-01

**Authors:** Doris Boeckelmann, Mira Wolter, Barbara Käsmann-Kellner, Udo Koehler, Lea Schieber-Nakamura, Barbara Zieger

**Affiliations:** 1 Department of Pediatrics and Adolescent Medicine, Division of Pediatric Hematology and Oncology, Medical Center, Faculty of Medicine, University of Freiburg, 79106 Freiburg, Germany; doris.boeckelmann@uniklinik-freiburg.de (D.B.); mira.wolter@uniklinik-freiburg.de (M.W.); lea.nakamura@uniklinik-freiburg.de (L.S.-N.); 2 Department of Ophthalmology, Saarland University Medical Center, 66421 Homburg, Germany; kaesmann@gmail.com; 3 MGZ—Medical Genetic Center, 80335 Munich, Germany; koehler@mzg-muenchen.de; 4 University Medical Center, Department of Neuropediatrics and Muscle Disorders, Faculty of Medicine, University of Freiburg, 79106 Freiburg, Germany

**Keywords:** Hermansky-Pudlak syndrome, HPS-11, bleeding tendency, hypopigmentation, oculocutaneous albinism, *BLOC1S5*

## Abstract

Hermansky-Pudlak syndrome (HPS) is a heterogeneous disorder combining oculocutaneous albinism (OCA) and a platelet function disorder of varying severity as its most prominent features. The genes associated with HPS encode for different BLOC- (biogenesis of lysosome-related organelles complex) complexes and for the AP-3 (adaptor protein-3) complex, respectively. These proteins are involved in maturation, trafficking, and the function of lysosome-related organelles (LROs) such as melanosomes and platelet δ-granules. Some patients with different types of HPS can develop additional complications and symptoms like pulmonary fibrosis, granulomatous colitis, and immunodeficiency. A new type of HPS has recently been identified associated with genetic alterations in the *BLOC1S5* gene, which encodes the subunit Muted of the BLOC-1 complex. Our aim was to unravel the genetic defect in two siblings with a suspected HPS diagnosis (because of OCA and bleeding symptoms) using next generation sequencing (NGS). Platelet functional analysis revealed reduced platelet aggregation after stimulation with ADP and a severe secretion defect in platelet δ-granules. NGS identified a novel homozygous essential splice site variant in the *BLOC1S5* gene present in both affected siblings who are descendants of a consanguine marriage. The patients exhibited no additional symptoms. Our study confirms that pathogenic variants of *BLOC1S5* cause the recently described HPS type 11.

## 1. Introduction

Hermansky-Pudlak syndrome (HPS, OMIM Phenotype Series—PS203300) is a rare heterogeneous disorder affecting maturation, trafficking, and the function of lysosome-related organelles (LROs) such as melanosomes and platelet δ-granules. HPS is thus characterized by oculocutaneous albinism (OCA) and a bleeding diathesis of variable severity. Additionally, cell type specific LROs may be affected, leading to pulmonary fibrosis (aberrant alveolar macrophage or mast cell function), to granulomatous colitis (intestinal granulomas and inflammatory cells), and immunodeficiency (cytotoxic T-cell-granules).

Since its first description in 1959 by Hermansky and Pudlak [1], knowledge of the clinical symptoms, underlying genes and pathomechanism of HPS has deepened over the last few decades. Next-generation sequencing in particular has increased the number of diagnosed patients, since molecular genetic analysis can assess a multi-gene panel much faster than gene-by-gene direct sequencing. Up until 2020, alterations in 10 genes had been reported to cause HPS type 1 to type 10; however, Pennamen et al. reported just recently pathogenic variants in the *BLOC1S5* gene (OMIM *607289) associated with a new type of HPS named type 11 (#619172) [2].

All HPS genes are involved in the biogenesis of four ubiquitously expressed multi-subunits complexes: BLOC-1 (biogenesis of lysosome-related organelles complex-1), BLOC-2, BLOC-3 and AP-3 (adaptor protein-3) [3].

The genes *DTNBP1, BLOC1S3, BLOC1S6* and *BLOC1S5* code for subunits of the BLOC-1 complex and are associated with the HPS-7, HPS-8, HPS-9 disorders and HPS-11, respectively. Main phenotypical presentations of the few patients described so far are OCA and a bleeding diathesis.

The *HPS3, HPS5* and *HPS6* genes code for the three subunits of the BLOC-2 complex [4], leading to HPS-3, -5, and HPS-6 [5,6,7,8]. Patients with a BLOC-2 deficiency often present with a milder phenotype such as OCA with variable hypopigmentation and moderate bleeding diathesis.

*HPS1* and *HPS4* code for the two subunits of the BLOC-3 complex. Patients with HPS-1 and HPS-4 are at risk of developing pulmonary fibrosis with an onset as young adults or middle age, respectively [9,10,11,12,13].

Defects of the adaptor protein-3 (AP-3) are due to alterations in the *AP3B1* (HPS-2) and *AP3D1* (HPS-10) genes. Interestingly, patients with an HPS-2 or HPS-10 defect suffer from immunodeficiency in addition to OCA and bleeding diathesis [14,15,16]. HPS-2 patients carry the risk of developing pulmonary fibrosis with an onset in childhood [17]. Right before HPS-11 was first described, Huizing et al. summarized the reports of patients with HPS types 1-10 worldwide in their mutation update [18].

The BLOC-1 complex contains eight subunits: pallidin, cappuccino, dysbindin, snapin, muted, BLOS1, BLOS2, and BLOS3 [19,20], with a subcomplex organization of pallidin-cappuccino-BLOS1 and dysbindin-snapin-BLOS2, respectively [21]. Besides the ability to sort proteins in mammalian cells, the BLOC-1 complex is involved in recycling of endosomes bringing together the actin and microtubule cytoskeleton [22].

Very few patients with defects in the subunits dysbindin (*DTNBP1*, HPS-7) [6,23,24,25,26], BLOS3 (*BLOC1S3*, HPS-8) [6,27,28,29], pallidin (*BLOC1S6*, HPS-9) [30,31,32,33,34] and muted (BLOC1S5, HPS-11) [2,35] have been described so far. However, reports of pathogenic variants in the *BLOC1S4* gene, which encodes the subunit cappuccino known to be associated with an HPS phenotype in mice, will likely follow [36]. Snapin and BLOS1 knockout mice are inviable [37,38].

In 2002, Zhang et al. showed in the muted mouse model that the gene encodes a protein involved in regulating vesicle trafficking. Mice with mutations in the muted gene (orthologue the human *BLOC1S5*) exhibit a Hermansky-Pudlak phenotype [39]. The human *BLOC1S5* gene encodes three transcripts: Isoform 1 (NM_201280.3; 2659 nucleotides) encoding for the longest protein (NP_958437.1; 187 aa), and Isoform 2 (NM_001199322.1; 2798 nt) and 3 (NM_001199323.1; 2565 nt) coding for NP_001186251.1 (123 aa) and NP_001186252.1 (90 aa), respectively (https://www.ncbi.nlm.nih.gov/; accessed on 17 August 2021). After the first description of two unrelated patients with homozygous pathogenic variants in the *BLOC1S5* gene by Pennamen et al., a patient from China was described with a homozygous nonsense variant in *BLOC1S5* [35]. These three reported HPS-11 patients presented with moderate OCA (creamy skin and lighter hair than their parents, nystagmus and retinal hypopigmentation) and mild to moderate bleeding diathesis.

Here we report the case of two brothers with a suspected HPS diagnosis for over a decade. However, a pathogenic/likely pathogenic variant in the direct sequencing of the candidate genes for HPS-1–HPS-8 had not been identified. Now next generation sequencing (NGS) using a multi gene panel finally identified a novel homozygous likely pathogenic variant in the *BLOC1S5* gene leading to a molecular genetic diagnosis of HPS-11.

## 2. Materials and Methods

### 2.1. Ethic Statement

This study was approved by our local ethics committee. Informed consent was obtained from the patients and/or their parents before genetic analysis was performed.

### 2.2. Patients

The patients (P1 and P2) are two brothers from Uzbekistan who presented with OCA and bleeding symptoms in our outpatient clinic about 10 years ago during a visit to Germany. The parents are related and have black hair and darker skin color than their 2 children. Both brothers presented with creamy skin and light-blond hair. The older brother’s ophthalmological features (P1) comprised nystagmus, retinal hypopigmentation, iris transillumination, optic nerve decussation anomalies on visual evoked potentials, strabismus, photophobia, and visual acuity of 20/200 in both eyes. The younger brother (P2) presented with nystagmus, ocular albinism, including retinal hypopigmentation, iris transillumination, photophobia, and visual acuity of 20/200 and 20/100 in the right and left eyes, respectively. P1 reported epistaxis lasting a few hours three to four times a year; circumcision did not trigger increased bleeding complications. His brother (P2) reported epistaxis twice a year, getting more severe over time; he suffered from extensive bleeding after a circumcision at the age of 5 years.

### 2.3. Platelet Count and Platelet Aggregometry Analyses

Platelet count was measured via an automated cell counter (Sysmex KX-21 N, Norderstedt, Germany). Platelet-rich plasma (PRP) and platelet-poor plasma (PPP) were obtained by centrifugation of citrate-anticoagulated blood samples. Using the APACT 4 (LABiTec, Ahrensburg, Germany), platelet aggregometry analyses were performed after stimulation with collagen (2 µg/mL; Takeda, Linz, Austria), adenosine diphosphate (ADP; 4 µmol/L; Sigma-Aldrich, St. Luis, MO, USA), epinephrine (8 µmol/L; Sanofi-Aventis, Frankfurt, Germany) and ristocetin (1.2 mg/mL; American Biochemical and Pharmaceutical Ltd., Frankfurt, Germany).

### 2.4. Flow Cytometry Analyses

Flow cytometry analyses were performed using FACSCalibur (Becton Dickinson, Heidelberg, Germany) [40]. Diluted PRP aliquots (5 × 10^7^ platelets/mL) were fixed and stained with FITC-labeled monoclonal surface antibody against CD41 (GPIIb/IIIa-complex), CD42a (GPIb/IX) and CD42b (GPIb) (Coulter, Immunotech, Marseille, France). FITC-labeled anti-VWF (Bio-Rad AbD Serorech, Puchheim, Germany) and Alexa Fluor 488-labeled anti-fibrinogen (Invitrogen, Waltham, MA USA) were used to stain the platelets. In the presence of 1.25 mM Gly Pro-Arg-Pro (Bachem, Bubendorf, Switzerland) diluted PRP (5 × 10^7^ platelets/mL) was stimulated with several concentrations of thrombin (0, 0.05, 0.1, 0.2, 0.5 and 1 U/mL; Siemens Healthineers, Marburg, Germany) to conduct the CD62 and CD63 expression analyses. The platelets were also stained with monoclonal FITC-labeled anti-CD62 (P-selectin) and anti-CD63 antibodies (lysosomal membrane associated glycoprotein 3, LAMP-3; Immunotech, Marseille, France).

### 2.5. Molecular Genetic Analyses

To extract genomic DNA from EDTA-blood, we applied standard procedures and the Blood and Cell Kit by Qiagen (Qiagen GmbH, Hilden, Germany). Panel sequencing (95 genes including all 11 HPS-genes) was performed in both patients using a custom designed Nextera Rapid Enrichment Kit (Illumina, San Diego, California/USA) followed by sequencing on a MiSeq (Illumina). SeqPilot (JSI medical systems) was used for data analyses. The variants were exported and filtered by allele frequency and serious consequences. We used supporting software ALAMUT^®^ (v.2.15), pathogenicity prediction (CADD; Combined Annotation Dependent Depletion), occurrence in population and disease databases (HGMD public version, Huizing HPS Mutation update [18]) to classify the variants. These analyses were conducted following the ACMG (American College of Medical Genetics) guidelines [41]. Sanger sequencing was performed for confirmation. *BLOC1S5* exon 2 and intronic boundaries were amplified using the following primers (F, forward; R, reverse): F- 5′- TCT CTT AGT GGG GAA GGG AGA GAG T-3′ and R- 5′-CCC TAG AGC AGG CAC CAG AAC T-3′. Array-CGH (comparative genomic hybridization) analyses were performed using the SNP array Infinium^®^ CytoSNP-850K (Illumina, San Diego, CA, USA) according to the manufacturer’s instructions.

## 3. Results

### 3.1. Platelet Aggregometry Revealed Impaired Platelet Aggregation and Platelet Flow Cytometry Showed a Delta-Granule Secretion Defect

P1 and P2 presented with normal platelet count (247 G/L and 249 G/L, respectively). Light transmission aggregometry revealed only slight impaired aggregation after stimulation with all agonists for P2, whereas P1 showed only slight altered aggregation after stimulation with ADP (Table 1).

**Figure 1:**
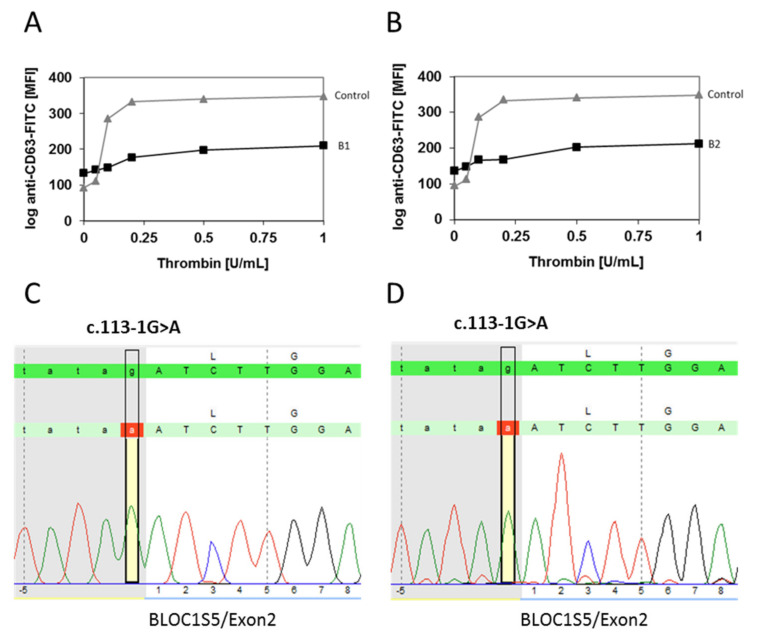
Upper panel: Platelet granule secretion after stimulation with thrombin (concentrations: 0, 0.05, 0.1, 0.2, 0.5, and 1.0 U/mL) for the two brothers using flow cytometry. Severely impaired δ-granule secretion indicated by reduced platelet CD63 expression in P1 (**A**) and P2 (**B**) compared to the healthy control. Data are expressed as logarithmic arbitrary units (logAU) of anti-CD63-stained unstimulated and thrombin-stimulated platelets. Lower panel: Sanger Sequencing for confirmation of *BLOC1S5*:c.[113-1G > A];[113-1G > A]. Reverse chromatograms for patient P1 (**C**) and P2 (**D**).

Interestingly, flow cytometry analysis showed severely reduced CD63 expression after activation with thrombin, indicating a delta-granule secretion defect in the platelets (Figure 1A,B).

### 3.2. NGS Identified a Novel Homozygous Likely Pathogenic Variant in BLOC1S5

Molecular genetic analysis using a multi-gene panel was performed for the brothers. Average sequencing depth over all genes for the two patients investigated was 99% for 20 × coverage and 98%/99% for 100 ×, respectively. The brothers shared a novel homozygous essential splice site variant c.113-1G > A in BLOC1S5 (NM_201280.2) confirmed via direct sequencing (Figure 1C,D). This variant is located at the acceptor splice site of intron 1. The consequence of this change is not predictable, but a skip of exon 2 is very likely. There was no patient’s mRNA available to prove the splice effect; however, the ALAMUT^®^ included splice defect prediction tools (MaxEnt., NNSPLICE, and SSF) predicted a splice defect (−100%). The CADD score is 34, a score of greater or equal 20 indicates the 1% most deleterious substitutions. This novel likely pathogenic variant is not listed in the gnomAD (v2.1) public database in either its homozygous or heterozygous state, or in dbSNP. Array-CGH analyses ruled out compound heterozygosity with a deletion and confirmed the most likely biallelic state of the variant. Overall ACMG pathogenicity criteria are: PVS1, PM2, PP3, PP4 = “Pathogenic”. Because we could not further characterize the splice defect, we classified the variant as likely pathogenic.

## 4. Discussion

Our study identified a novel homozygous essential splice site variant located at the acceptor splice site of intron 1 in *BLOC1S5* in two brothers presenting with an HPS phenotype. Novel variants in a gene in which loss of function (LOF) is a known disease mechanism and that lead to serious consequences for the encoded protein (null variant: nonsense, frameshift, canonical ± 1 or 2 splice sites, initiation codon, single- or multi-exon deletion) are considered, with very strong evidence (PVS1), to be pathogenic. All three human BLOC1S5 transcripts include exon 1–2 and therefore are affected by the splice site variant. Because the brothers live in Uzbekistan, we could not obtain further material for cDNA sequencing or Western blot analysis of platelets to further investigate the splice defect. However, the alteration (c.133-1G > A) at the canonical acceptor splice site (AG → AA) is considered to lead to abnormal splicing. The software SeqPilot’s copy number analysis revealed no alteration indicating a potential deletion of *BLOC1S5* exons. Because we did not have DNA from the related parents for segregation analysis, we performed array-CGH analyses to exclude a potential allelic deletion masking homozygosity. Moreover, NGS analysis of the two brothers’ DNA did not detect pathogenic/likely pathogenic variants in any of the other HPS genes in a homozygous or heterozygous state. In addition, NGS did not reveal pathogenic/likely pathogenic variants in the genes associated with Griscelli syndrome type 1-3 (*MYO5A*, *RAB27A*, *MLPH*) or Chediak–Higashi syndrome (*LYST*).

Regarding the three reported patients so far with HPS-11 (a 20-yr-old female originating from French Flanders, a 39-yr-old female originating from Slovenia, and a male patient from China), the authors identified either a deletion of two exons or a 1-basepair deletion leading to a frameshift/protein truncation or a premature STOP codon [2,35]. Together with the canonical splice site variant identified in our study patients, there are now 5 HPS patients identified with *BLOC1S5* null variants (multi-exon deletion, nonsense, frameshift, canonical ± 1 or 2 splice sites). All alterations lead to serious consequences for the protein.

The patients reported so far suffer from hypopigmentation of the skin and hair, ophthalmologic symptoms such as photophobia, and poor visual acuity. Two patients’ bleeding diathesis was mild: bleeding after minor traumas in the Chinese patient; and the French Flanders patient’s bleeding diathesis was not apparent until the molecular genetic diagnosis was made. The latter patient had initially reported no signs of bleeding diathesis; however, after counseling her history revealed easy bruising, epistaxis once or twice a year, and gingival bleeding. The female patient originating from Slovenia suffered from epistaxis, easy or unexplained bruising, and excessive blood loss after deliveries, surgery, and dental extraction. Menorrhagia improved via contraception. She also reported abdominal pain, dyspnea, and recurrent infections (pneumonia, herpes, conjunctivitis) [2]. The two male patients in our study presented with OCA and mild to moderate bleeding diathesis similar to the French Flanders patient.

Molecular diagnosis is essential for genetic counseling. Furthermore, comprehensive diagnostics help to improve the treatment after trauma-induced bleeding and prevent post-surgical bleeding.

Patients with defects in other BLOC-complexes, e.g., in the BLOC-3 complex or AP3-complex, can develop pulmonary fibrosis, granulomatous colitis, and immunodeficiency. Most of the few patients with a BLOC-1 deficiency do not develop such features. However, individual patients have been reported with suspected granulomatous colitis (HPS-7) [26] or lymphocyte-predominant Hodgkin’s lymphoma (HPS-8) [29], an association previously described in HPS-2 patients [42]. Milder features like mild primary immunodeficiency or gastrointestinal problems have been reported for a few patients as well. Two unrelated HPS-9 patients (from Italy and Pakistan) share the same homozygous nonsense mutation in *PLDN* (HGNC approved gene symbol: *BLOC1S6*:c.232C>T (p.Q78*)). Badolato et al.’s Italian patient presented a mild primary immunodeficiency coinciding with leukocytopenia and recurrent cutaneous infections. Their patient’s anomalies were not as prominent as those in HPS-2 patients [30]. Their 17-year-old patient did not suffer hemorrhagic episodes, and platelet aggregation tests were thus normal. They conducted no further investigation of the platelet delta granule secretion. The Pakistani patient, a 4-yr-old female, suffered from OCA, photophobia, nystagmus, prolonged bleeding, and platelet dysfunction. The girl also suffered gastrointestinal distress including, nausea, vomiting, abdominal pain, and diarrhea [34]. Mild leukocytopenia with no history of recurrent infections has been reported for another HPS-9 patient from Japan [33]. The granule secretion defect in leucocytes generally seems to be so mild that patients with BLOC-1 deficiencies stand out primarily because of their OCA. The bleeding severity in patients with a BLOC-1 deficiency varies, and females, in particular, may have increased problems because of menorrhagia or extensive post-partum bleeding) [26]. We summarized all reported cases of HPS-7, HPS-8, HPS-9, and HPS-11 in Table 2.

The brothers in our study, now 19 and 16 years old, have not developed any signs of immunodeficiency, pulmonary or intestinal problems so far. However, their diagnosis of suspected Hermansky-Pudlak syndrome in 2012 led to a better understanding of their symptoms, and therefore, to better care for them (skin and eye care, awareness of a bleeding disorder concerning possible surgery). The most serious problem was the untreated visual defect. Tinted corrective glasses, 20% for inside use and 80% for outside, helped to reduce glare sensitivity. NGS now led to the diagnosis HPS type 11. This will help to better assess the disease not only for these patients, but for assessing different types of the Hermansky-Pudlak syndrome as well. In this ultra-rare disease, every patient report is important, so that knowledge can be deepened with respect to the HPS-11 phenotype. If a genetic diagnosis of a BLOC-1 deficiency is made, patients should also be carefully examined for slight defects in lysosome-related organelles in other cell types in order to characterize these rare diseases more precisely. A description of these brothers’ phenotype and genotype will help us gain more insights in the only recently described HPS-11.

## Figures and Tables

**Table 1:** Platelet aggregometry analyses for P1 and P2.

Agonist	Max. Aggregation [%] P1	Max. Aggregation [%] P2
Collagen (2.0 µg/mL)	83	70 desaggregation
Ristocetin (1.2 mg/mL)	85	87 desaggregation
ADP (4 µmol/L)	69 desaggregation	77 desaggregation
Epinephrine (8 µmol/L)	72	64

Normal max. aggregation: >70%.

**Table 2:** Pathogenic variants identified in BLOC-1 genes associated with HPS type 7, 8, 9, and 11.

Gene (Transcript) Disease	gDNA/mRNA	Amino Acid	Variant Type	dbSNP (rs)/ClinVar	Ethnic Background (Age, Gender)	References
*DTNBP1* (NM_032122.4) HPS-7	c.177G>A	p.Trp59*	Nonsense	rs727502866	Caucasian (77y, F)	Lowe et al. (2013), [26]
	c.307C>T	p.Gln103*	Nonsense	rs104893945	Portuguese (48y, M), Paraguayan (6y, M)	Li et al. (2003), [25]
					Portuguese (siblings: 26y, M; 56yr, F)	Bryan et al. (2017), [24] Bastida et al. (2019), [23]
					Portuguese (18y, F)	Bastida et al. (2019), [23]
	c.771_774del	p.Asn257Lysfs*13	Indel	-	1 case	Lasseaux et al. (2018), [6]
	c.1017_1020del	p.Glu340Profs*44	Indel	rs759180894	Argentinian (M);compound heterozygous with c.307C>T	Unreported ^1^
*BLOC1S3* (NM_212550.3) HPS-8	c.131C>A	p.Ser44*	Nonsense	rs281865115	Iranian (6y, M)	Cullinane et al. (2012), [27]
	c.338_341del	p.Leu113Argfs*15	Indel	SCV001192839	Brazilian (10y, M)	Pennamen et al. (2021), [29]
	c.385_403del	p.Ser129Glnfs*90	Indel	SCV001192837	1 case	Lasseaux et al. (2018), [6]
					North African (15, M) and his affected sibling	Pennamen et al. (2021), [29]
	c.444_467del	p.Gln150_Ala157del	Indel	rs754841982SCV001192838	1 case	Lasseaux et al. (2018), [6]
					Portuguese (12y, M)	Pennamen et al. (2021), [29]
	c.448del	p.Gly150Argfs*75	Indel	rs281865116	Pakistani (6 familial cases)	Morgan et al. (2006), [28]
*BLOC1S6* (NM_012388.4) HPS-9	c.148G>T c.351dup	p.Glu50* p.Ile118Tyrfs*10	Nonsense Indel	-	Chinese (6y, M)	Liu et al. (2021), [31]
	c.200C>G c.319_320delinsAT	p.Ser67* p.Glu107Met	Nonsense Indel	-	Syrian (4m, F)	Michaud at al. (2021), [32]
*BLOC1S6* (NM_012388.3) HPS-9	c.232C>T	p.Gln78*	Nonsense	rs201348482	Italian (17y, F),	Badolato et al. (2012), [30]
					Pakistani (4y, F)	Yousaf et al. (2016), [34]
	c.285_286dup	p.His96Leufs*22	Indel	-	Japanese (52y, F)	Okamura et al. (2018), [33]
*BLOC1S5* (NM_201280.2) HPS-11	Chr6(GRCh37):g.8023117_8042179del, deletion of exons 3and 4		Large deletion, copy number loss	VCV000813287.1	French Flanders (20, F)	Pennamen et al. (2020), [29]
	c.113-1G>A		Splice site	-	Uzbekistan (siblings: 19y, M; 16y, M)	This study
	c.181del	p.Val61*	Nonsense	rs774712389	Chinese (unknown, M)	Zhong et al. (2021), [35]
	c.345del	p.Val116Serfs19*	Indel	-	Slovenia (39y, F)	Pennamen et al. (2020), [29]

Abbreviations: y, years; M, male; F, female, *, termination. ^1^ listed in the NIH HPS cohort referred by Dr. Rosenzweig and in the Mutation update from Huizing et al. (2020).
